# Drought and Heat Stress in Cool-Season Food Legumes in Sub-Tropical Regions: Consequences, Adaptation, and Mitigation Strategies

**DOI:** 10.3390/plants10061038

**Published:** 2021-05-21

**Authors:** Venugopalan Visha Kumari, Anirban Roy, Roshni Vijayan, Purabi Banerjee, Vivek Chandra Verma, Arpita Nalia, Madhusri Pramanik, Bishal Mukherjee, Ananya Ghosh, Md. Hasim Reja, Malamal Alickal Sarath Chandran, Rajib Nath, Milan Skalicky, Marian Brestic, Akbar Hossain

**Affiliations:** 1 Department of Agronomy, Faculty of Agriculture, Bidhan Chandra Krishi Viswavidyalaya, Mohanpur 741252, India; visha.venugopal@gmail.com (V.V.K.); anirbanneelroy@gmail.com (A.R.); itsmepurabi1@gmail.com (P.B.); arpita.nalia6@gmail.com (A.N.); madhusri.bckv@gmail.com (M.P.); bishalmukherjee@gmail.com (B.M.); ananya.ghosh0193@gmail.com (A.G.); rejahasim92@gmail.com (M.H.R.); sarathagri@gmail.com (M.A.S.C.); rajibbckv@yahoo.com (R.N.); 2 AINP (Arid Legumes), Division of Pulses, Regional Agricultural Research Station—Central Zone, Kerala Agricultural University, Pattambi, Melepattambi P.O., Palakkad Kerala 679306, India; roshnivij@gmail.com; 3 Defence Institute of High Altitude Research, Chandigarh 160002, India; vivekverma95@gmail.com; 4 Department of Botany and Plant Physiology, Faculty of Agrobiology, Food, and Natural Resources, Czech University of Life Sciences Prague, Kamycka 129, 165 00 Prague, Czech Republic; skalicky@af.czu.cz; 5 Department of Plant Physiology, Slovak University of Agriculture, Nitra, Tr. A. Hlinku 2, 949 01 Nitra, Slovakia; 6 Department of Agronomy, Bangladesh Wheat and Maize Research Institute, Dinajpur 5200, Bangladesh

**Keywords:** heat stress, drought stress, legumes, adaptation, mitigation strategies

## Abstract

Drought and heat stress are two major abiotic stresses that challenge the sustainability of agriculture to a larger extend. The changing and unpredictable climate further aggravates the efforts made by researchers as well as farmers. The stresses during the terminal stage of cool-season food legumes may affect numerous physiological and biochemical reactions that may result in poor yield. The plants possess a good number of adaptative and avoiding mechanisms to sustain the adverse situation. The various agronomic and breeding approaches may help in stress-induced alteration. The physiological and biochemical response of crops to any adverse situation is very important to understand to develop mechanisms and approaches for tolerance in plants. Agronomic approaches like altering the planting time, seed priming, foliar application of various macro and micro nutrients, and the application of rhizobacteria may help in mitigating the adverse effect of heat and drought stress to some extent. Breeding approaches like trait-based selection, inheritance studies of marker-based selection, genetic approaches using the transcriptome and metabolome may further pave the way to select and develop crops with better heat and drought stress adaptation and mitigation.

## 1. Introduction

Food legumes or cool-season legumes are protein-rich and are commonly called ‘*poor man’s meat*’. They help to meet the diverse demand for food, fiber, and fodder in several agricultural systems [1,2]. They are known for their completeness in a balanced diet and also form an integral part of the vegetarian diet. Pulses like broad beans (*Vicia faba*), lupin (*Lupinus* spp.), lentils (*Lens culinaris*), chickpeas (*Cicer arietinum*), grass peas (*Lathyrus sativus*), dry peas (*Pisum sativum*), and the common vetch (*Vicia sativa*) are included in the list of cool-season pulses [3]. They are named so mainly because of the cooler conditions they require for vegetative growth. Though cool-season food legumes are of less importance in world production and consumption, they form a very imperative component in the Indian diet.

Food supply, as well as nutritional security, has been highly challenged by rising temperatures. Small farmers are severely impacted by heat stress affecting their crops [4]. More so than global temperature rise, local temperature increases are of much concern as they can drastically affect crop production. The prevalence of high temperatures is anticipated to amplify in the near future [5]. More flowers per plant enhance yields per plant and ultimately better production. However, various stress cause an imbalance in the production of the reproductive organ affecting the yield. Under various abiotic stresses, among which heat and drought are a major threat nowadays, hamper the reproductive ability of crops. Plant stress can be broadly grouped into biotic stress and abiotic stress. The stress caused by any living organisms such as pathogenic bacteria, fungus, viruses, nematodes, insects, and phenerogamic plants are known as biotic stresses, and stress caused by the physical environment like temperature, moisture, relative humidity, sunshine, etc., are known as abiotic stresses. Drought and heat are the major abiotic stresses that have been reported to reduce crop productivity and yields and affect food security. Considering the current change in climate, we expect an increase in the severity of abiotic stresses, which may impact global food security.

The escalating variability in crops is expected to result in plants more sensitive to heat and drought stress [6]. Changes in temperature thresholds, even by a single degree beyond the threshold level, are sure to affect the production potential of crops [7]. Heat stress can cause physiological, morphological, anatomical, and biochemical changes in the growth and development of a plant that may finally affect the yield of the crop [7,8]. Though the stresses have a negative effect from the germination to the reproductive stage, the latter is considered more serious as it affects productivity to a larger extent, and even stress at the reproductive stage invites root diseases [9].

Plants are known for their genetic potential in allowing them to adapt to an unfavorable environment. However, it varies depending on the crop, variety, and the management option they are exposed to and also the quantum of exploitable genetic variation present in its germplasm [10]. As mentioned earlier, even a slight variation from the normal condition may affect yields; it is very important to understand the stress complex and act accordingly. This review presents a zest of the effect of heat and drought stress, its effect on plants, and mitigation strategies. 

## 2. Drought and Heat Stress in Cool-Season Food Legumes

Drought stress takes place if the air temperature is high and soil and atmospheric humidity are low; disproportionate water uptake and evapotranspiration from the soil results in this condition [11]. Plant growth through cell division, enlargement, and differentiation is impaired along with mitosis and cell elongation [12]. The stress hinders cell enlargement owing to loss of turgidity. The loss of turgidity results in smaller and lesser leaves, thus reducing the photosynthetic area [13]. The effect of drought stress is generally impulsive as various factors, including rainfall patterns, water holding capacity of the soil, and evapotranspiration may influence it. Drought stress also affects growth; photosynthesis assimilates partitioning and hampers yields [14,15].

When soil and or air temperature increase beyond a threshold level for a stipulated time is known as heat stress, which may affect crop growth. It may result in various visual symptoms like sun scorching, leaf burn, leaf discoloration, and senescence [16,17]. The duration of which the crop is at a high-temperature matters a lot under heat stress. Reproductive growth is largely affected under high temperatures. A temperature ≥ 30 °C, can result in poor pollen viability, pollen shedding, poor pollen germination and growth, and decreased pollen elongation [18]. 

Though both stresses have differences in their occurrence, most of the time, they are observed together, causing a lot more negative effects on plant growth and yield than what they cause alone. For this very reason, both the stress needs to be considered [19]. Severe yield losses have been reported by researchers in many crops. Some cool-season food legumes affected by drought and heat stress are given in Table 1.

## 3. Change in Climate: A Major Reason for Heat and Drought Stress in Cool-Season Food Legumes

The high pace increase in the global population and drastically changing climate is challenging global food security to a large extent [26]. The sustainability of an agricultural system is at high risk due to climate change. The reduction in annual rainfall, along with the erratic pattern of monsoon rains, possesses a high threat of frequent drought around the world [27]. Though a surge in temperature is reported positive in some cooler parts and crops, the larger impact is depressing [28]. India being an agricultural nation, the vulnerability of the changing climate is of larger magnitude than in other nations. It is very clear that among all the stresses, drought and heat stress are the major ones for agricultural production [29]. Both these stresses have a larger impact in reducing the yield and causing a negative impact on farmer livelihoods than others. 

Food legumes can acclimatize to a wide range of soil and climatic conditions, and hence, could be a component in adaptation strategies to the changing climate. As these cool-season food legumes (CSFLs) are grown in the *Rabi* season, the major hurdle a farmer faces is for its timely sowing. Change in weather is forcing farmers to begin to grow their crops late (late *Kharif*) as there is a shift of monsoon rains from June–July to August–September. Apart from this, the growers prefer long-season *Kharif* crops. These long-season crops often keep the land occupied till the end of November, delaying the sowing of the subsequent CSFLs. The sowing may be further delayed due to excessive drought due to untimely and unpredicted rains [30].

### 3.1. Consequences of Heat and Drought Stress on Food Legumes

Cool-season food legumes (CSFL) are more susceptible to temperature variation than warm-season food legumes. Increasing the threshold level always results in the growth and productivity of legumes [31]. In most cases, even an increase of one degree in temperature will matter a lot and is considered as heat stress in these plants [32], which has serious implications for growth and biochemical function [7]. 

Though any small increase in temperature is considered a stress, the impact largely depends on the level and duration of the exposure. Larger impacts have been observed in the physiological processes of crops, such as photosynthetic reserves, nitrogen assimilation [33], protein catabolism, and the accumulation of the end products of lipid peroxidation [34,35]. It also affects the phenology of crops, including effects on photosynthetic machinery, which are affected due to non-robust photosystems; in most cases, the duration gets shortened, resulting in poor yields [36]. Stress affecting CSFLs during their reproductive stage has been reported in legumes such as chickpea [37,38], field peas [39], and others.

The critical temperature for heat tolerance in chickpeas has been reported as being much higher than the tolerances of other legume crops like faba beans, field peas, and lentils [40]. A temperature increase beyond 30 °C causes stress in chickpeas. It may lead to an early start to the reproductive stage, affects the physiology of flowers, and may result in a lower seed set [41]. Similar results have been reported by GrossandKigel [42] in beans. In the common bean, as reported by Nakano et al. [43], a change in night and day temperature of 27/32 °C during sporogenesis may result in reduced pollen viability, pollen sterility, and low pod numbers and seed sets. Maheshwari et al. [44] reported on the detrimental effect of high temperature on photosynthesis, respiration process, cell water relations, and membrane permanence, compatible solute adjustment, and the accumulation of antioxidant compounds, etc. Threshold temperature for several cool-season food legumes are listed in Table 2.

High-temperature stress causes oxidative damage by the formation of ROS (reactive oxygen species) in plants [45]. ROS affect cell functioning by damaging membranes, lipids, and proteins. Oxidative damage further leads to membrane instability. A higher lipid and protein peroxidation was reported under drought stress conditions compared with normal conditions in field peas [46]. Membrane stability further affects chloroplasts and thus photosynthesis and assimilate transportation. The outcome is largely a reduction in yields and quality. 

Stomatal closure is the first and foremost response to any stress to avoid water loss. This may be a response to lower leaf water potential [47] or lower atmospheric humidity [48]. Further closing results in CO_2_ intake, which leads to oxidative damage and no assimilation. Almost all the important and essential nutrients are taken up by the roots along with water. Prevailing stresses reduce the movement of nutrition and water and retards growth. The other interesting fact reported under drought conditions is the response of stomata to ABA [49]. However, the intensity of response to ABA may be different between plant species [50]. 

**Table 2:** Threshold temperature for cool-season food legumes.

Legumes	Temperature	Reference
Chickpea	15–30	[51]
Faba bean	25	[52]
Lentil	15–33	[53]
Lupins	20–30	[54]
Field pea	20–34.3	[55]

### 3.2. Impact on Seed Setting and Yield

Researchers and farmers are concerned about seed setting and yield, a stage of growth that deals with transport of source from leaves to reproductive organs, and deals with processes related to the storage of nutrients [56]. High-temperature stress in legumes results in larger yield losses, mainly because of poor seed setting [57,58,59]. The impact of high temperature on seed setting of cool-season food legumes is given in Table 3. Seed filling time is shortened due to high temperatures, resulting in a reduction in crop duration and yield [60]. The reduction in seed filling time may reduce seed weight [61]. The crop experiencing stress due to higher temperatures (30–35 °C) during the reproductive phase can result in a reduction of legume yield [59,62].

## 4. Mitigation Strategies

### 4.1. Agronomic Strategies

#### 4.1.1. Application of Plant Nutrients as a Foliar Spray

Under heat stress, high water status can be attained by various macronutrients and micronutrients. The macronutrients and micronutrients like potassium, calcium, boron, selenium, and manganese activate various physiological and stomatal functions in plants [67]. Apart from the stress amelioration, foliar spray of micronutrients can also improve the nutritional quality of crops [68].

Zinc is another micronutrient known for its metabolic and regulatory functions [69], which also plays a pivotal role in the reproductive phase of the crop. Zinc deficiency in the soil affects the development of anthers [70], pollen viability [71], and inhibits pollen-stigma interaction [72]. Another micronutrient, Iron (Fe), is important for various biochemical pathways in plants [73,74]. The impact of boron deficiency on assimilate partitioning may greatly influence the ability of plants to cope with other unfavorable environmental conditions such as soil water deficits and low supplies of other nutrients. Boron plays an important role in the reproductive growth of plants [75,76]. The role of boron for pistil development and pollination to fertilization has also been reported by many researchers. In oilseed rape, Xu et al. [77] observed the arrested development of ovules and embryo sacs in flowers from low B plants. They also noted unusual development of stigmatic papillae. Another study has recorded almost double the yield with the foliar spray of B+Fe at 0.5% than the treatment with no foliar spraying in lentils, which were sown late and experienced both heat and drought stress [78].

#### 4.1.2. Plant Growth Regulators

Plant growth regulators are also known for their effects in mitigating stress. They help in regulating hormone transduction pathways [79]. The role of auxins during heat stress responses has recently attracted attention, and there is some strong experimental evidence regarding their role in thermo-protection. Auxins have been implicated in imparting thermo-tolerance to reproductive components (anthers) [80]. Salicylic acid, cytokinin GA, and ABA have been reported for their usefulness in mitigating the adverse effect of drought stress (Table 4) and temperature stress by increasing the water potential and sink capacity [81,82].

#### 4.1.3. Seed Priming

Seed priming is an improved agronomic intervention involving restricted hydration of seeds before sowing [89] to influence the performance of a crop on a long-term basis [89,90]. Priming develops tolerance to stress during the seedling stage [91] as well as throughout the growing season of a crop [92]. Priming induced faster germination and uniform emergence [93,94,95,96,97,98,99], allowing farmers to cope with the time lost in drought [100]. Compared to the unprimed seeds, generally, primed ones give rise to more robust seedlings with extensive root systems and complete their life cycle earlier. Many researchers have established the fact that seed priming is a great technique for accelerating crop growth and flowering for better yields in rice fallows [101,102]. In a way, these properties promote a healthier stand establishment of a crop by expanding the area for soil water and nutrient uptake. On the other hand, seed priming also promotes leaf area enlargement for capturing more solar radiation, thereby accelerating photosynthetic activity and consequent increment in yield potentials of crops [103]. All these advantages may prove seed priming to be an outstanding approach to escape terminal heat and drought stress [104,105,106]. In fact, priming is a very affordable practice to alleviate the adversities of abiotic stress in crop plants [107,108,109,110,111], before or after the germination of seed and stand establishment [112,113,114,115]. The reason behind the better preparedness of primed seeds for possible stresses may be the activation of the response of antioxidant systems through priming, and Se and Si application protects against drought stress if applied during priming [89].

Priming of seeds with KH_2_PO_4_, Na_2_HPO_4_, etc., or water have been found to develop drought withstanding ability in crops [116]. BABA (β-amino butyric acid), a non-protein amino acid, has been reported to impart resistance against abiotic stress in plants [117,118,119,120]. This may be a result of hormonal activity such as salicylic acid, abscisic acid, and ethylene [119,121]. The literature also contains reports regarding priming seeds with bacterial inoculum, which stimulated the enzymatic activity in stress-induced plants with respect to ROS scavenging property [122,123,124,125].

#### 4.1.4. Planting Method

Winter pulses are more susceptible to drought, especially during the later part of the growth stages, if not supplemented with lifesaving irrigation or grown under drought conditions with residual soil water. Relay sowing of cool-season legume crops after rice reduces the negative impact of terminal drought and heat stress during the reproductive phase of the pulse crop by overlapping part of its early growth stage with the previous crop. It has been reported by Gangwar et al. [126] and Kar and Kumar [127] that legumes grown after wet season rice with reduced or minimum tillage give a higher yield. The relay sowing of lentils is done in some areas of eastern India by broadcasting the seed in the standing rice crop 15 days prior to its harvesting without any tillage, which grows well by utilizing the residual soil water present in the field [127,128].

The major components of conservation agriculture, i.e., reduced tillage practices and surface retention of crop residues, imparts significant direct and indirect effects on drought conservation, reduction in soil temperature, and runoff losses. It may help in balancing soil hydrothermal properties. Conservation tillage practices, along with mulching, have a very positive impact on the growth and productivity of pulses (chickpeas). Soil with hardpans needs deep tillage to conserve soil moisture. Layek et al. [128] and Ghosh et al. [129] reported that adopting suitable planting techniques (relay cropping), along with crop residue retention in rice fallows, helps mitigate terminal drought in lentils by reducing evaporation loss hence conserving soil moisture. In dryland areas, chickpea and field pea can be grown in FIRB (Furrow irrigated raised bed system) to conserve soil water as well as enhancing productivity [130]. To downturn, the adverse effect of drought and heat stress on cool-season legumes needs the choice of proper resource conservation practices, tillage methods, cropping systems, selection of appropriate pulse varieties (varietal diversification), etc.

#### 4.1.5. Planting Time

Time of sowing is the most important nonmonetary input that has significant effects on crop growth, phenological development, insect-pest, weed dynamics, and crop productivity. Sowing date is an important determinant of crop yields. Delayed sowing results in rising temperature and longer photoperiod, and consequently, rapid vegetative growth that hastens crop maturity. Terminal heat and drought stress restrict vegetative growth and branching, leading to forced maturity and lower yields [131]. A yield reduction of 20%–70% of yield reduction occurs in cool-season pulses like chickpeas, field peas, faba beans, and lentils due to flower drop and pod abortion caused by rising of temperature above 25 °C [132]. Crop establishment, growth, development, and the environment during seed set are all influenced by the time of sowing [133]. Heat stress induces flowering before the plant has grown sufficiently to bear a good crop. Rabi greengram can be sown up to the end of December, and this is practiced in the southern part of India, where the winters are not severe [134].

In the case of late sown cool-season legumes, the increase in leaf temperature causes decreased leaf water potential and relative water content, which ultimately reduces the photosynthetic rate of the crop [14]. As the rate of transpiration and plant growth are highly related and affected by appropriate planting time, this will be one of the most vital agronomic practices to be adopted for getting higher yield, specifically under heat-stressed situations [135]. In principle, delays in sowing beyond the optimum dates result in a progressive reduction in reproductive growth, i.e., flowering, pod formation, and ultimately, crop yield.

#### 4.1.6. Role of Plant Growth-Promoting Rhizobacteria and Arbuscular Mycorrhizal Fungal Inoculation in Mitigating Stress

Plant growth-promoting rhizobacteria (PGPR) have a very important role in mitigating drought stress in legumes [136]. The mechanism by which it deals with drought stress includes mechanisms such as phosphorus solubilization, organic acids, nitrogen fixation, production of siderophores, and production of enzymes such as ACC deaminase [137,138]. The efficiency in water use efficiency through hormonal signaling has also been reported [139]. Phytohormones like gibberellins, auxins, cytokinins, ABA, and ethylene can also be synchronized by PGPR [140]. In a study in lentils, it was found that application of *Pseudomonas putida* enhanced nodulation and plant growth that resulted in better drought resistance [141]. Under drought stress situations, arbuscular mycorrhizal fungi (AMF) help in water and nutrient uptake [142]. They increase the levels of osmoprotectants and antioxidant potential thus, decreasing lipid peroxidation [143]. AMF plays a vital role in improving soil structure and soil water retention ability through the formation of soil aggregates [144].

### 4.2. Genetics and Genomics Approaches

#### 4.2.1. Genetics/Breeding Approaches

**Figure 1:**
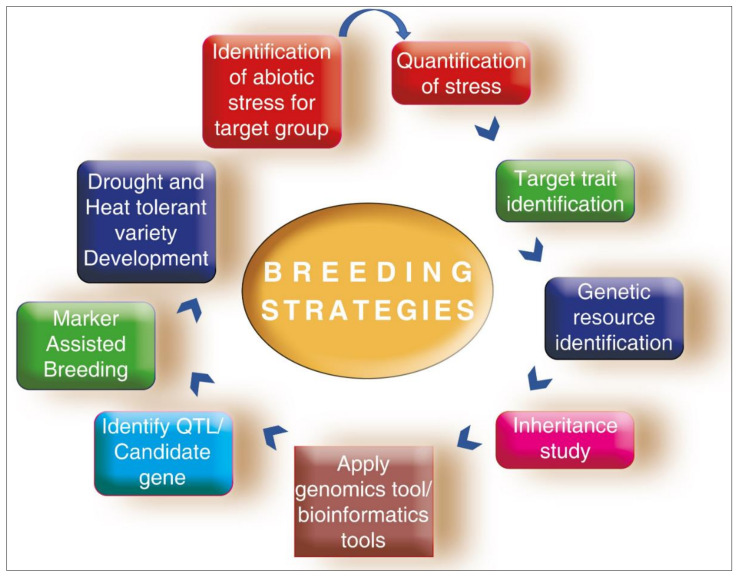
Breeding strategies deployed for cool-season legume breeding.

Breeding strategies deployed for cool-season legume breeding are shown in Figure 1.

##### Target Traits for Breeding Drought and Heat Tolerance

Stress causes changes in plant physiology that cause reproductive malfunctioning. Stress sensing through signal transduction quickly hampers several metabolic changes, especially in reproductive organ development, loss of several dehydrogenase activities, sucrose metabolism, starch synthesis and partitioning, phytohormonal sensitivity, etc. For traits like early flowering, early vigor in lentils can be used as a drought escaping mechanism. Less cell membrane injury and early seedling growth, i.e., quick establishment, are key traits for drought tolerance in lentils [145]. Lesser shoot/root ratios can be used as selection criteria for drought tolerance. Vegetative to reproductive stages are vulnerable to stress irrespective of crops, including cereals, with effects varying over wider ranges [146]. Deep root, photosynthesis-related traits, and osmotic adjustments are major selection criteria that can be used for pigeon pea for drought tolerance screening [147]. A profuse rooting system, early maturity with reasonable yield during drought, are widely used traits for selection during drought stress for chickpeas [148].

##### Inheritance Studies

Identification of genetic elements for drought tolerance can be exploited using large-scale germplasm screening, including wild species and cultivated exotic and indigenous collections. A wide potentiality for drought tolerance ability has been exploited in wild lentils [149]. To increase the speed of breeding for drought tolerance through germplasm screening, variability can be created in chickpeas by inducing mutations [150]. Regarding genetics, inheritance studies for target traits are important, as conventional plant breeding strategies take a long time to develop a suitable genotype selection from segregating population; hence, marker-assisted technologies are needed to increase the speed of the election cycle and improve genetic gains. During the development of generation segregation, population mapping is key for identifying the affected genes and the nature of their effects, and their QTLs and target genes. Drought tolerance traits are governed by a complex set of polygenes, but in cowpeas, drought resistance is reported to be governed by a single dominant gene [151].

##### Traits and Their Susceptibilities

Changes in night temperature affect optimum biomass [152], random heat and drought cause drastic yield reductions [153]. Combined heat and drought stress cause hampered photosynthesis rates [154]. Unusually prolonged winter spells lead to shorter reproductive durations and less reproductive partitioning, including leaf physiology, which has the effect of lowering photosynthate production. Early heat stress causes untimely flowering and leads to drastic yield reductions; pre-flowering heat stress induces lower numbers of flowering buds, even basic cellular homeostasis and chlorophyll damage occurs due to unusual photooxidative damage due to PSI activation; breeding is targeted at inhibiting this activation process [155]. Post flowering heat stress causes flower drying. Moisture deficit stress at the early vegetative stage reduces branching and flowering in stunted seedlings; even moisture deficit stress at the reproductive stage causes forced maturity. Excessive drought at the early stage and reproductive stage causes early senescence and forced maturity. Even in the case of high-temperature rises during vegetative and reproductive stages drastically hampers the yield capacity of plants.

#### 4.2.2. Genomics Approaches

Drought is the most complex target trait for breeding as, during drought stress, some other abiotic stress or biotic stress may be associated, making it more complex. Mechanisms of drought and its implication for breeding have been well studied in a few crops, and that information can be used using a genomic tool for other crops [156]. Under drought and heat stress, from perception to signal transduction to physiological adjustment towards plant tolerance by minimizing the detrimental effect on reproductive fecundity, which is controlled by a set of interacting loci under QTL, and understanding that quantitative loci through genomic approaches, is key [157]. Even whole-genome resequencing programs have helped to identify a large number of SNPs and copy number variants have been used in association studies in various crops. Sequence analysis using various bioinformatics applications for exploiting undeciphered features of gene-like structures studies can be performed for identifying the genetic causes of tolerance under changing environmental conditions [158].

##### Mapping of Genomic Regions for Complex Traits

As roots first sense drought, root traits like root-length density, root biomass, and root length have been used in chickpea breeding for terminal drought tolerance [159,160]. For drought tolerance QTL identification, a QTL-Hotspot has been identified in a RIL population generated for mapping of drought tolerance [161], covering a large set of main effects and epistatic QTLs. 

##### Candidate Gene Discovery

The exploitation of available genetic potential has been utilized in the past as a common strategy [162]. Since long candidate gene identification followed by validation can help crop improvement [163], in silico analysis from available genomic information in the EST database can be used in crop breeding through identifying various EST-based marker development and genotyping [164]. Several high throughput NGS platforms for deciphering unexploited genomic information and also various third-generation genomic technologies have been used in breeding programs for understanding the genetics of target strategies [165,166]. Utilization of common genetic information for various crops is still under development as in several crops, where genomic information is not sufficiently strong that it can overcome syntenic crop information as *Cicer*, *Lens*, *Vicia*, and *Medicago* have 40% genomic similarity [167]. cDNA library generation for particular stress can yield strong genomic information regarding the transcript response to that stress [168]. 

Abiotic stress is a complex mechanism as it is controlled by a number of genetic factors and their interaction leads to tolerance. Trait-based mapping of the abiotic stress plays an important role in accelerating the speed and accuracy of breeding [10]. To find the entire story, major signaling genes are identified that actually perceive the stress and regulate metabolism, ultimately leading to considerable yields under stress. In this context, the candidate gene approach is linked to the genomics approach for genetic gain under stress conditions. Mammalian AMPK (AMP-activated protein kinase) and budding yeast SNF1 have a plant ortholog, the SNF1 (sucrosenon-fermenting1)-related protein kinases1 (SnRKs1). These are evolutionarily conserved kinases. In response to declining energy levels, these metabolic sensors are activated, i.e., when glucose and sucrose amount reduces during stress. As complex trimeric SnRK1, after sensing stress in the cell, ceases carbohydrate translocation to ensure expenditure of available energy to that particular area. When SNF1/AMPK/SnRK1 kinases are activated, they trigger reprogramming of transcriptional and metabolic activities and, in turn, restore energy homeostasis, which thereby develops tolerance to adverse conditions. This is achieved by general repression of anabolism and induction of catabolic processes. The SNF1/AMPK/SnRK1 kinases typically function as a heterotrimeric complex that is controlled by multiple mechanisms and requires phosphorylation of a conserved activation loop residue for activity. The complex is made up of βandγ regulatory subunits and an α-catalytic subunit [169]. The SnRK1 heterotrimeric complex exists in several isomeric forms, acts as a metabolic regulator for nutrient deficiency in plants. By the characterization of the seeds of *M*. *trancatula*, isomeric forms of β (MtAKIN β1-β4) and γ(MtAKINβγ, MtSNF4β, MtAKINγ) regulatory subunits were identified, which helped to gain insight into the development of responses to stress conditions in the plants. Transcripts accumulation was found to be different in vegetative as well as seed tissues and also modulated differently with the imposition of stress and during the germination process.

Transcription factors (TFs) play the major roles for downstream gene up and down-regulation upon exposure to stress for short or long-term stress via interaction through cis-acting elements [170]. The role of the bZIP transcription factor has been studied in drought tolerance, and WRKY and NAC TFs have also been widely studied and exploited for their extensive role in cereals crops [171]. An important class of TFs, DREB (Dehydration responsive element binding)/CBF (C-repeat binding factors), which bind to DRE (Dehydration responsive element) functions, providing multiple stress tolerance, and growth-dependent MADS-box TFs manipulate flowering behavior [172]. 

It generally functions more in an ABA-independent manner through DRE/CRT (C-repeat) cis-acting elements and AP2/ERF (Apetala2/Ethylene Responsive Factor) DNA binding domain. So understanding the mechanism of heat stress dissecting the new area in lentils to make the crop more tolerant to heat has been unexplored until now, although an experiment using *Medicago trancatula* revealed upregulation of the MtCBF4 gene during drought, salinity, and cold stress [173]. As most abiotic stress tolerance mechanism overlaps, finding more in lentils is another input that could lead to genetic improvements. Several major candidate genes that have been exploited for drought tolerance, including the LEA protein group, which have been validated through a transgenic approach, which can be used for genetic polymorphism study of these genes in breeding programs in terms of sequence-based polymorphisms and expression [174]. Adaptation under elevated temperature is responded to by changing flowering gene expression, including series of FT genes, which enhances adaptation and response can be useful in candidate gene-based breeding.

##### Markers for Genetic Dissection

A wide range of markers are used for searching for polymorphisms, mapping, and marker-assisted breeding, including RAPD and RFLP [175]. Genomic information in food legumes is not as strong as in other cereal crops. Syntenic information and cross-species transfer of SSR has been practiced, following strong genomic information that facilitated the development of crop-specific SSR markers [176]. A large number of SSR markers, including EST SSR, have been used for ‘*QTL Hotspot*’ identification in chickpeas [176]. Interaction of simple screening along with biochemicals protecting cells and marker development for QTLs have been the most comprehensive approach since the improvement of genomics technologies [177]. 

### 4.3. The Transcriptome and Metabolome

This strategy is now a common genomic approach for gathering initial information for any crop regarding any abiotic stress for orphan crops as well as well exploited crops in terms of genomic information. The related abundant transcripts which are responsive to stress can be identified through this technique covers a whole-genome picture for that particular trait. Even basic pathways that are linked with the stress response and tolerance can be identified, which provides a basic understanding for breeders of the traits involved and can inform breeding directions under such conditions. The drought tolerance gene has been dissected using spatiotemporal gene expression analysis using NGS in chickpeas [164]. Genes responsive to drought tolerance in lentils have been exploited through transcriptome analysis [177] for seedling drought tolerance. Complete metabolic profiling under control as well as stress condition gives an idea regarding metabolic changes and metabolic regulations under various stresses for various pathways that are major determining factors for yield components, i.e., complete development of the reproductive part of the crop. The complete metabolic picture is the initial clue regarding the candidate gene for target trait-based breeding.

**Figure 2:**
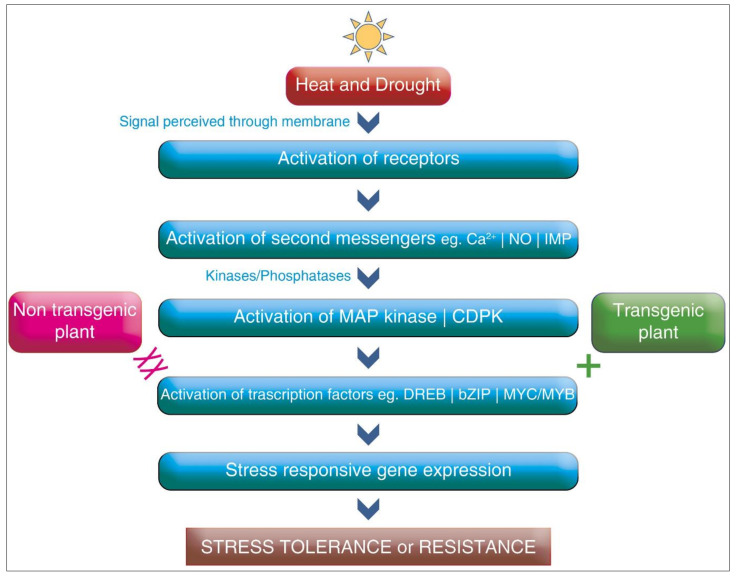
The basic mechanism of the transgenic approach during stress.

The advancement of transgenic crop approaches, especially gene-based technology, appears to be the most valuable tool for resistance against heat and drought stresses. The basic mechanism of the transgenic crop approach for heat and drought stress is shown in Figure 2. The tools of biotechnology help us alter the genetic makeup of crops to protect them against devastating abiotic stress conditions. The identification of the specific genes which make the organisms resistant to abiotic stress and transfer them to crops from any organism, even from different species, can be achieved with the advances made in biotechnology [178]. The engineering of stress tolerance genes that encode antioxidants, compactable solutes, and growth regulators has been the major emphasis. For developing drought-tolerant crops, genetic engineering has been utilized for improving the gene expression of glycine betaine in higher plants, which encodes for two enzymes, choline monooxygenase and beta aldehyde dehydrogenase [179]. The genes regulated by the DREB and AREB proteins are also being studied for drought stress tolerance in several crops [180]. Overexpression, as well as underexpression, of certain genes in transgenic plants, have been studied for drought tolerance. Kudo et al. [181] reported that the overexpression of *DREB1A* and *OsPIL1* genes improved drought tolerance in transgenic plants.

Sometimes heat stress impacts the electron transport system, and some studies have found that reduced electron flow between photosystems indirectly protects plants from photoinhibition [182,183]. Gosal et al. [184] reported the importance of the accumulation of LEA proteins in drought tolerance. The genes involved in the expression of LEA proteins, which help plants in maintaining the cell membrane structure and ionic balance under drought stress, have been developed [185]. The concept of the transgenic approach elucidates different candidate genes involved in response to complex abiotic stress in legume plants. *Agrobacterium*-mediated transformation or biolistic methods are some of the methods by which transformation has been achieved for developing transgenic legumes. Past studies suggest that improved plant performance in drought conditions without having any negative impact on yield has been achieved with the incorporation of resistance genes into various genomes [183]. Legume plants that confer abiotic stress resistance have been genetically engineered that encode enzymes involved in the modification of membrane lipids and the biosynthesis of osmoprotectants and late embryogenesis proteins [186]. 

**Figure 3:**
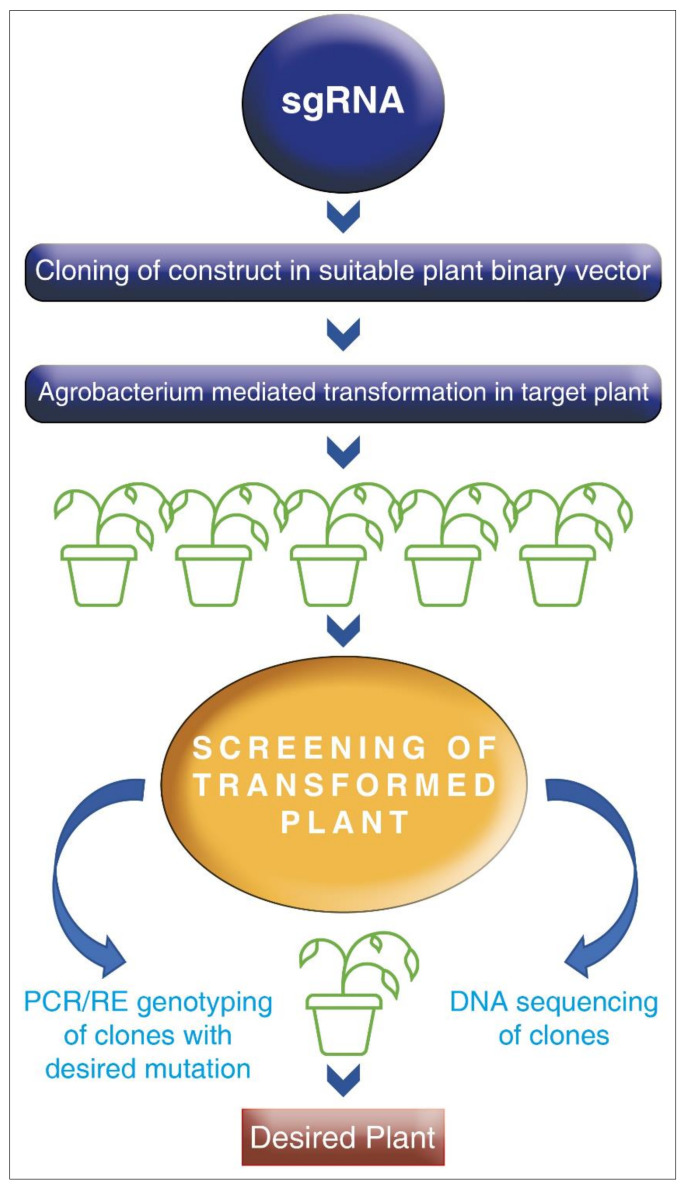
Schematic representation of genome editing (GE) using Cas9/sgRNA.

In response to complex stress environments, genes belonging to the AP2/ERF family and DREB transcription factors have a pivotal role in plant growth and development [187]. Genetic recombination and random mutagenesis are the major means of improvement of variability; however, it is a laborious process. With the rampant growth of the human population, in order to keep pace with rising food demands, crop improvement has to be advanced with newer technologies. One of the unique technologies for genetic manipulation, CRISPR/Cas9, has opened a new arena for the engineering of any genomic sequence with any target gene of interest more efficiently. The engineered CRISPR contains a guide RNA or single guide RNA (gRNA or sgRNA) and a CRISPR-associated endonuclease (Cas protein). The gRNA is a short synthetic RNA with a ~20 nucleotides spacer and scaffold sequence necessary for Cas-binding. The gRNA base pairs with an RNA target, orienting bounds protein to carry out a site-specific cleavage, ligation, or modification reaction. Thus, one can change the genomic target of the Cas protein by simply changing the protein sequence present in the gRNA CRISPR/Cas9 is a more straightforward technology that does not fall under the regulatory monitoring mechanism as available for GM crops. This technology helps in the development of non-genetically modified plants, which are suitably edited with desired traits and can contribute to enhancing crop production under any stress (Figure 3).

A new study revealed that CRISPR/Cas9 mediated genome editing could be an essential technology to develop crops with improved tolerance to abiotic stresses [188]. In Leguminous crops, only a few studies have adopted CRISPR/Cas9 for editing drought tolerance-related genes. The first achieved success using CRISPR/Cas9-mediated gene editing was in soybeans, where a single sgRNA for transgene (*bar*) and six sgRNAs targeting various sites of two genes (*GmSHR* and *GmFE12*) were used [189]. CRISPR/Cas9 gene editing has huge potential for developing elite cultivars of legumes with durable and higher climate resilience by targeted and precise mutagenesis. Abiotic stress tolerant legumes developed through transgenic methods are listed in Table 5. Some of the genetic resources for cool-season legume crops in available gene banks are given in Table 6.

## 5. Conclusions

The sustainable use of genetic resources for food and agriculture is the foundation of many of the climate change adoption strategies. The need for more farmer-friendly approaches and new genotypes adapted to stress is the need of the hour. The management approaches are not new; however, the time for implementing each approach and understanding crop responses is important. The breeding of adaptive traits is required for increasing the resilience of crops to current climate change conditions to help sustain productivity. To fast track the genetic gains with respect to stress in food legumes with the speed of climate change for crop improvement approaches like genomic assisted breeding, next generation of genomic assisted breeding approaches, data management, breeding programs, pre-breeding, and trait discovery are needed. The application of omics for understanding the molecular basis and plant response to temperature stress will pave the way for developing plants with better tolerance to temperature stress, ultimately leading to nutritional and food security.

## Figures and Tables

**Table 1:** Yield losses caused by drought and heat stress in some major food legumes.

Legumes	Stress	Yield Losses (%)	References
Chickpea	Heat and drought	40–45	[20]
Lentil	Heat and drought	57	[21]
Soybean	Drought	73–82	[22]
Cowpea	Drought	29	[23]
Faba bean	Drought	40	[24]
Field pea	Drought	21–54	[25]

**Table 3:** Impact of high temperature on seed setting of cool-season food legumes.

Legumes	Temperature (°C)	Impact on Seed Setting	Reference
Chickpea	34/19	Epidermis wall thickening of anthers, ovule abnormality, pollen germination, and receptivity	[40]
Lentil	32/20	Pod abortion reduced flower no. shortened flowering period, pollen germination, pollen tube length, pod length	[63,64]
Lupins	33/28	Ovule abortion	[65]
Field pea	27/36	Pollen germination, tube length, and pod growth	[66]

**Table 4:** Protective effects of plant growth regulators in cool-season food legumes.

Legumes	Protectants	Protective Effects	References
Chickpea	ABA	More growth, less oxidative damages decreased MDA and H_2_O_2_ contents.	[83]
Wintergreen gram	Salicylic acid	Its endogenous level in heat-stressed mungbean plants enhances antioxidant enzyme activities to impart thermotolerance.	[84]
Chickpea	Salicylic acid and Putrescine	Increased leaf proline content, greater lipid peroxidation, and accelerated antioxidant enzymes (CAT, APOX, POD, and SOD) activity.	[85]
Faba bean	Exogenious Gibberelic acid	Balanced activity of osmoprotectants, nutrients, antioxidant defense mechanism, and phytohormones.	[86]
Lentil	Exogenious proline and Betain	Upregulation of homeostasis in lentils under stress conditions, modulation of glutathione S-transferase, glyoxalase I, and GSH content with a decrease in GSSG and H_2_O_2_ levels, thereby reducing the toxic impacts of reactive oxygen species and the methylglyoxal detoxification system.	[87]
Field pea	24-Epibrassinolide and Thiourea	Enhanced contents of relative leaf water, total chlorophyll, and soluble sugars in response to drought stress.	[88]

**Table 5:** Abiotic stress resistance in legumes.

Transgenics	Gene Incorporated	Source	Stress Mitigated	References
Soybean	*P5CR*	*Arabidopsis thaliana*	Heat and drought stress	[190]
Chickpea	*P5CSF129A*		Increase in proline synthesis	[191]
Chickpea	*DREB1A*	*Arabidopsis thaliana*	Drought tolerant	[187]
Soybean	*LOSS/ABA3*		Drought tolerant	[192]
Cowpea	*VuNCED1*	*Vigna unguiculata*	Drought tolerant	[193]
Soybean	*GmRACK1*	*Glycine max*	Drought tolerance during vegetative growth	[194]
Soybean	*AtABF3*	*Arabidopsis thaliana*	Enhance drought tolerance	[195]
Chickpea	*DREB2A, MYB, WRKY,bZIP,XPB1*		Enhance tolerance to drought	[196]
Chickpea	*P5CSF129A*		Increase in proline synthesis	[191]
Chickpea	*DREB1A*	*Arabidopsis thaliana*	Drought tolerant	[187]
Soybean	*LOSS/ABA3*		Drought tolerant	[192]
Cowpea	*VuNCED1*	*Vigna unguiculata*	Drought tolerant	[193]
Chickpea	*CaP5CS*	*Cicer arietinum*	Increases proline synthesis under water stress	[197]
Alfalfa	*Mn-SOD gene*	*Nicotinia plumbaginifolia*	Drought tolerant	[198]
Soybean	*Gm(DREB2, FDL19, SK1, BIN2, NAC, DREB, ZIP)*	*Glycine max*	Enhance drought tolerance	[199,200,201,202,203]

**Table 6:** Genetic resources of cool-season food legume in major gene banks.

Crop	Gene Bank	Number	Source
Lentil	Major gene banks of World	58,405	[204]
Chick Pea	>30 countries of the world gene banks	>80,000	CGIAR Gene bank database
Lathyrus	ICARDA gene bank	3315	[205]
Pea	16 Major gene banks of world having more than 1000 entry	57,341	[206]
Faba Bean	ICARDA gene bank	15,386	[205]

## Data Availability

Not applicable.
